# Similar support for three different life course socioeconomic models on predicting premature cardiovascular mortality and all-cause mortality

**DOI:** 10.1186/1471-2458-6-203

**Published:** 2006-08-04

**Authors:** Maria Rosvall, Basile Chaix, John Lynch, Martin Lindström, Juan Merlo

**Affiliations:** 1 Department of Health Sciences, Lund University, Malmö University Hospital, Malmö, Sweden; 2 Department of Clinical Sciences, Lund University, Malmö University Hospital, Malmö, Sweden; 3 Research Unit in Epidemiology, Information Systems, and Modelisation (INSERM U707), National Institute of Health and Medical Research, Paris, France; 4 Department of Epidemiology, Biostatistics and Occupational Health, McGill University, Montreal, Canada

## Abstract

**Background:**

There are at least three broad conceptual models for the impact of the social environment on adult disease: the critical period, social mobility, and cumulative life course models. Several studies have shown an association between each of these models and mortality. However, few studies have investigated the importance of the different models within the same setting and none has been performed in samples of the whole population. The purpose of the present study was to study the relation between socioeconomic position (SEP) and mortality using different conceptual models in the whole population of Scania.

**Methods:**

In the present investigation we use socioeconomic information on all men (N = 48,909) and women (N = 47,688) born between 1945 and 1950, alive on January, 1^st^,1990, and living in the Region of Scania, in Sweden. Focusing on three specific life periods (i.e., ages 10–15, 30–35 and 40–45), we examined the association between SEP and the 12-year risk of premature cardiovascular mortality and all-cause mortality.

**Results:**

There was a strong relation between SEP and mortality among those inside the workforce, irrespective of the conceptual model used. There was a clear upward trend in the mortality hazard rate ratios (HRR) with accumulated exposure to manual SEP in both men (p for trend < 0.001 for both cardiovascular and all-cause mortality) and women (p for trend = 0.01 for cardiovascular mortality) and (p for trend = 0.003 for all-cause mortality). Inter- and intragenerational downward social mobility was associated with an increased mortality risk. When applying similar conceptual models based on workforce participation, it was shown that mortality was affected by the accumulated exposure to being outside the workforce.

**Conclusion:**

There was a strong relation between SEP and cardiovascular and all-cause mortality, irrespective of the conceptual model used. The critical period, social mobility, and cumulative life course models, showed the same fit to the data. That is, one model could not be pointed out as "the best" model and even in this large unselected sample it was not possible to adjudicate which theories best describe the links between life course SEP and mortality risk.

## Background

**Figure 1:**
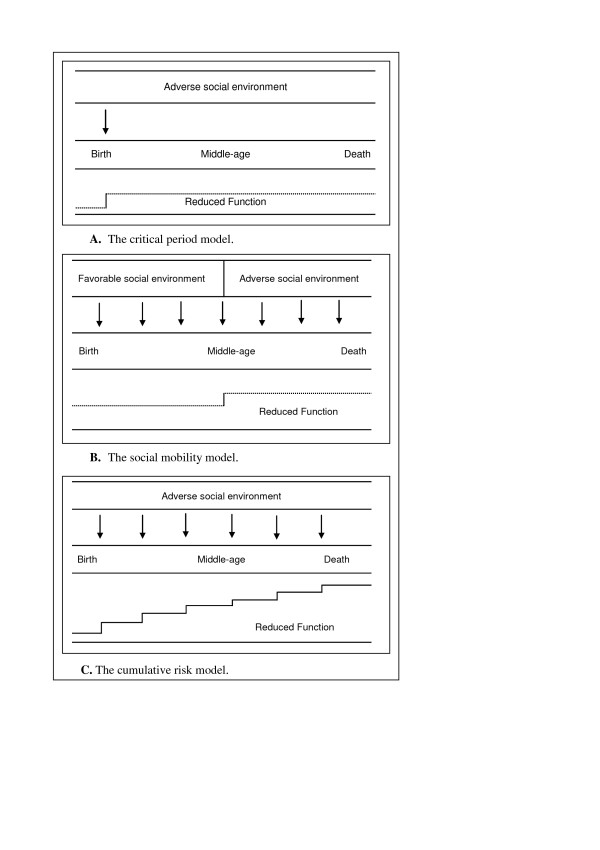
**Crude schematic illustration of three life course socioeconomic models**. A. Critical period model: focuses on the importance of an independent effect of social exposure during a specific sensitive period in life having lasting effects on adult health; B. Social mobility model: focuses on the importance of change in social position to adult health. C. Cumulative model: focuses on the importance of accumulation of risk during the life course to adult health.

There has been growing interest in a temporal perspective on socioeconomic differences in the development of disease. Several studies have shown an association between low socioeconomic position (SEP) and mortality using multiple indicators of SEP over the life course [1-13]. Different conceptual models for the impact of the social environment on adult disease have been presented and have been grouped into at least three broad conceptual models: the critical period, social mobility, and cumulative life course models [14-16]. The critical period model (Figure 1A) focuses on the importance of an independent effect of social exposure during a specific sensitive period in life, having lasting effects on adult health [14-16]. The fetal origins hypothesis formulated by Barker and Osmond in the 1980s was based on this model [17]. Several studies have shown an association between early life SEP and mortality [1,5-7,9,12,18], while other studies could find no such association [2,3]. The social mobility model (Figure 1B) focuses on the importance of change in SEP to adult health [14-16], while the cumulative risk model (Figure 1C) focuses on accumulation of risk during the life course [14-16]. Social mobility theories hypothesize that SEP mobility across the life course impacts adult health, although the causal direction of these associations is not completely evident [16]. There are studies that have shown an increased mortality risk among the upwardly mobile [19], but also studies that have shown a decreased mortality risk [2,11]. The cumulative risk model assumes that risks to adult health gradually accumulate as the number, duration and severity of exposure increase [14-16]. Several studies have shown a cumulative effect of SEP during the life course on adult mortality risk [4,5,8,10-12].

Each model implies somewhat different interventions for avoiding premature mortality. For example, from a preventive perspective, it may be good to know whether exposure to low SEP is more important to adult health during certain periods in life and less important during others, or whether the risk to adult health gradually accumulates as the number, duration and severity of exposure to low SEP increase. However, the relative importance of each of these models is hard to interpret since the studies are often based on diverse populations and settings. Taking a life course approach to epidemiology [15] means attempting to synthesize previous approaches (i.e., the critical period, social mobility, and cumulative risk models). In a recent review by Pollitt et al [16], evaluating the evidence for models of life course socioeconomic factors and cardiovascular outcomes, it was concluded that analyses utilizing multiple life course designs within the same study offer the best approach to testing which theories best describe the links between life course SEP and cardiovascular (CVD) risk. Few studies have specifically investigated the importance of the different conceptual models in the same setting [14] and none were in samples of the whole population, where selection bias is minimized.

The purpose of the present study was to gain a more complete picture of the relation between SEP over the life course and cardiovascular and all-cause mortality in the whole population of Scania as the study population. We wanted to take a broad life course approach using three conceptual models, i.e., to study critical periods of SEP, the cumulative effect of SEP over the life course, and the effect of social mobility on adult premature cardiovascular and all-cause mortality. A similar framework was used in a Swedish population-based case-control study by Hallqvist et al. [14], investigating three conceptual models (the critical periods, accumulation, and social mobility models) in relation to incident first events of myocardial infarction among men aged 53–70.

Moreover, instead of excluding those outside the workforce from the analyses or categorizing them according to their latest or longest held occupation, as is often done in epidemiological studies, we chose to specifically study the importance of being outside the workforce during different periods in life using the same conceptual models as used for those inside the workforce. To the best of our knowledge, such an approach has not been used before, even though it has been argued that individual health problems are likely to inhibit entrance into the workforce or cause mobility out of it [20]. In an earlier study [21], those categorized as being outside the workforce were shown to have higher mortality rates with regard to CVD, cancer, psychiatric diseases, and external causes compared to those inside the workforce. This was true, even though those outside the workforce were a heterogeneous group and included, for example, students who would be expected to have relatively low mortality rates, as well as housewives and disability pensioners.

In the present investigation, we used socioeconomic information on all men (N = 48,909) and women (N = 47,688) born between 1945 and 1950, alive on January, 1^st^,1990, and living in the Region of Scania, in Sweden. Focusing on three specific life periods (childhood at age 10–15, and adulthood at age 30–35 and age 40–45), we examined the association between SEP and 12-year risk of premature cardiovascular mortality and all-cause mortality. In the analyses, we wanted to discriminate the independent effect of SEP in each specific period, as well as the effect of accumulation of low SEP (i.e., having a manual SEP as well as being outside the workforce) and change in SEP/workforce participation within and between generations.

## Methods

### Study population

With approval and assistance from Statistics Sweden and the Center for Epidemiology (Swedish National Board of Health and Welfare), a 43-year (1960–2003) longitudinal database, the Longitudinal Multilevel Analysis of Scania (LOMAS), has been assembled, which includes all the inhabitants in the county of Scania (about one million). The present study is based on a sub-cohort of the large LOMAS database, of 48,909 men and 47,688 women, as outlined above (see 'Background').

For every individual, we obtained information on causes of death from the Swedish Causes of Death Register [22] at the Centre for Epidemiology, The National Board of Health and Welfare, [23]. Statistics Sweden [24] provided information on the composition of the household as well as individual demographic and SEP (i.e., occupation) from the Swedish National Censuses performed in 1960, 1980 and 1990. The response rates of these censuses were 99 %, 98% and 98 %, respectively. A unique ten-digit personal identification number, assigned to each person in Sweden for their lifetime, was used for record linkage between the different registers. From the Housing Census in 1960, we also received information on crowded housing, defined as more than two inhabitants per room not counting the kitchen and one more room (e.g., the lounge).

The project was approved by the Regional Ethical Committee. We include those individuals in the study population, for whom data were available on SEP from all three age periods (N = 80,649). Out of those with available data on SEP in 1990, there were 15, 170 individuals with lack of data on either SEP in 1960 or on SEP in 1980.

### Measure of socioeconomic position

#### Whole population

Data on occupation, provided by Statistics Sweden [24], yielded information on the head of the household's primary occupation (in married couples i.e., the man's occupation) when the subject was 10–15 years old, as well as information on the subject's own occupation at ages 30–35 and 40–45. Job titles and work tasks formed the basis for classification into socioeconomic index (SEI) groups, according to the criteria of Statistics Sweden [25]. SEI classifications take into consideration the educational background needed to qualify for a particular job, additional employment prerequisites, job responsibility levels, and specific duties to be performed. Due to the fact that the classification scheme was somewhat less detailed in the census 1960 than in the censuses of 1980 and 1990, where similar occupational categories were used, we chose to focus on broad occupational categories. The SEI groups were therefore combined into five categories, namely, (1) non-manual employees (e.g., engineers with university degrees, college teachers, registered nurses, computer operators, secondary school teachers, office assistants, salespeople, secretaries); (2) manual workers (e.g., auto mechanics, metal workers, construction workes, factory workers, check-out assistant, waiters, janitorial staff), (3) self-employed persons (owners of businesses), (4) farmers; and (5) those inside the workforce for whom information on occupation was missing (unclassified). Subjects categorized as being outside the workforce included mainly homemakers, those who were unemployed, students and disability pensioners.

### Social mobility

#### Subjects having either manual or non-manual SEP at the three stages in life

Due to the unclear hierarchical relation to the other socioeconomic categories, subjects who were self-employed, farmers and persons with unclassified occupational status were excluded from the analyses on social mobility among those inside the workforce. Consequently, the analyses are based on data on 41,164 individuals having a manual or non-manual SEP at all three periods in life, i.e., 51 % of the study population. Intergenerational social mobility was defined as having a different SEP in childhood and in adulthood. It was defined as upward, downward or socially stable when comparing childhood SEP with the subject's own occupation at age 30–35 and at age 40–45. Subjects who were stable non-manual in childhood, at age 30–35 and at age 40–45 were used as the reference category.

Intragenerational social mobility was defined as having a different SEP at age 30–35 and at age 40–45. It was defined as upward, downward or socially stable when comparing the subject's own occupation at age 30–35 with the occupation at age 40–45. Subjects who were stable non-manual at age 30–35 and at age 40–45 were used as the reference.

#### Whole population

Intergenerational social mobility into and out of the workforce was defined as having a different category of workforce participation in childhood than in adulthood. Subjects who were stable inside the workforce in childhood (defined by workforce participation of the household), as well as at ages 30–35 and 40–45 were used as the reference. Intragenerational social mobility into and out of the workforce was defined as having different workforce participation at age 30–35 than at age 40–45. Subjects who were stable inside the workforce at ages 30–35 and 40–45 were used as the reference.

### Cumulative risk

#### Subjects having either manual or non-manual SEP at the three stages in life

A cumulative measure of SEP during childhood and adulthood was taken by means of a life course socioeconomic position (LCSEP) score, computed among individuals classified as having manual or non-manual SEP at the three periods in life (n = 41,164; 51 %). The LCSEP score ranged from 0 to 3 since it was calculated by summing SEP values (i.e., non-manual employees were given 0 points and manual workers were given 1 point) at ages 10–15, 30–35 and 40–45. Subjects whose childhood SEP was categorized as non-manual and who had a non-manual occupation at ages 30–35 and 40–45 were used as the reference group.

#### Whole population

Furthermore, a cumulative measure of workforce participation i.e., whether an individual was inside or outside the workforce, was taken by means of a score ranging from 0 to 3. Subjects categorized as being inside the workforce included the SEI-groups mentioned above. Individuals categorized as being outside the workforce included mainly homemakers, the unemployed, students and disability pensioners. In this scale, we combined the workforce participation of the head of the household during childhood and the subject's own workforce participation at ages 30–35 and 40–45 (i.e., those categorized as being inside the workforce were given 0 points and those categorized as being outside the workforce were given 1 point). Therefore, a cumulative measure of workforce participation equal to zero means that the individual was inside the workforce in all the three periods and a value of three that the individual was outside the workforce in all the three periods. Subjects whose head of the household during childhood was categorized as being inside the workforce and who themselves were inside the workforce at ages 30–35 and 40–45 were used as the reference group.

### Description of the use of three life course models

Table 1 includes a description of the use of three conceptual life course models based on SEP (i.e., having a manual or non-manual SEP) at three periods in life (ages 10–15, 30–35 and 40–45), while Table 2 includes a description of the use of three conceptual life course models based on workforce participation at the same age periods.

### Measurement of cardiovascular and all-cause mortality

Information on mortality in the LOMAS was obtained by record linkage with the Swedish Causes of Death Register [22]. The study population was followed with regard to all-cause mortality as well as cardiovascular mortality. Contributing or underlying causes of death were coded in accordance with the 8^th^, 9^th ^and 10^th ^version of the International Classification of Diseases (ICD), with codes  390–459 (ICD 8th and 9th version) or I00 to I99 (ICD-10th version) for cardiovascular disease.

### Statistical methods

Each individual was followed from January 1^st^, 1991, until December 31^st^, 2002, or death.

Cox proportional hazards models were used to estimate the ratios of hazard rates of cardiovascular and all-cause mortality between different socioeconomic groups over the follow-up period.

Hazard rate ratios (HRR) were estimated for each model (i.e., the critical period (1), social mobility (2), and cumulative risk models (3)) in relation to cardiovascular and all-cause mortality, using SPSS computer software (version 11.0). Mortality was investigated in relation to (1) childhood SEP at age 10–15, SEP at age 30–35 and SEP at age 40–45; (2) change in SEP, measured as: (a) mobility between manual and non-manual SEP within and between generations and (b) mobility into and out of the workforce within and between generations, using the same age periods and (3) the cumulative exposure to: (a) manual vs. non-manual occupation and (b) being outside vs. inside the workforce using the same three age periods. The HRRs were presented as age-adjusted stratified by sex. We used the Akaike Information Criterion (AIC) in SAS (version 9.1) to examine the model fit for each of the three life course models [26]. The lower the AIC, the better the fit of the model. Since it may be desirable to find the least complex model that best fits to the data, the AIC combines a measure of complexity and a measure of fit to identify the model that describes the data in the most efficient way.

## Results

There were 814 cases of cardiovascular death (578 men and 236 women) and 2,385 cases of death from all causes (1,391 men and 994 women). The unadjusted rate of cardiovascular mortality was 1.2 per 1,000 person-years for men and 0.5 per 1,000 person-years for women. The corresponding rates for all-cause mortality were 2.9 per 1,000 person-years for men and 2.1 per 1,000 person-years for women.

### Critical period

#### Whole population

Table 3 presents the HRRs of cardiovascular and all-cause mortality by SEP during the three age periods, i.e., childhood SEP at age 10–15, and adulthood SEP at ages 30–35 and 40–45, in Swedish men. At all three periods in life, having a manual SEP was associated with an increased risk of future mortality compared with having a non-manual SEP. Those outside the workforce generally showed the highest mortality risk at all three age periods. For example, men outside the workforce at age 40–45 had an HRR for cardiovascular disease and all-cause mortality of 4.9 (95 % Confidence interval (CI): 3.8, 6.3) and 4.9 (95 % CI: 4.2, 5.7), respectively. A similar pattern of association was seen in women (Table 4). After mutual adjustment, the HRR for mortality was reduced for each period. Among men, there were still independent effects of having a manual SEP at ages 30–35 and 40–45 with regard to cardiovascular and overall mortality. Among women, such effects could be seen for age 10–15 with regard to cardiovascular mortality. The strongest independent effect among those outside the workforce was seen at age 40–45, in both men and women.

### Social mobility

#### Subjects having either manual or non-manual SEP at the three stages in life

Table 5 presents the prevalences of the eight possible trajectories. More than half of the study population included in the analyses were socially stable, i.e, had the same SEP in all three periods in life. While having a manual SEP at all three periods in life was the most common trajectory in men, having a father in a manual occupation and having a non-manual occupation in adult life was the most common trajectory in women. Intergenerational downward social mobility was more common in men than in women, while intergenerational upward social mobility was more common in women. Irrespective of SEP in childhood, intragenerational upward social mobility was more common in men than in women, while intragenerational downward social mobility was about as common in men as in women.

**Figure 2:**
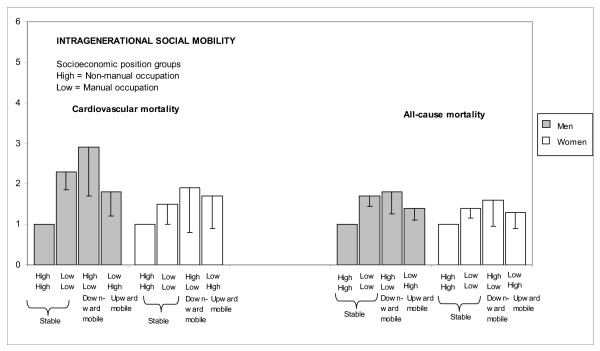
Age-adjusted HRRs for cardiovascular and all-cause mortality, by intragenerational socioeconomic mobility in Swedish men and women. Only subjects having either a manual or non-manual SEP at the three stages in life were included in the analyses. The different categories at each bar denotes socioeconomic position (SEP) at different periods in life – age 30–35 and age 40–45. Those stable non-manual at age 30–35 and at age 40–45 were used as the reference. The lower border of the 95 % confidence interval (CI) is marked.

Figure 2 shows the association between intragenerational social mobility and cardiovascular as well as all-cause mortality. The exact figures, with 95 % confidence intervals, can be seen in Table 6. Compared with subjects who were stable non-manual at the two stages in life, subjects who were stable manual showed a higher cardiovascular and all-cause mortality risk, in both men and women. Socially downward mobile men had an increased cardiovascular and all-cause mortality risk compared with men who were stable non-manual. This was also seen in women, however, the HRR for cardiovascular mortality did not reach statistical significance. Socially upwardly mobile men showed a higher mortality risk compared with men who were socially stable non-manual. This association, though weaker, was also seen in women. As shown in Table 6, a similar pattern of association was seen with regard to intergenerational social mobility.

#### Whole population

Table 7 shows social mobility into and out of the workforce. Being inside the workforce at all three periods in life was the most common trajectory in both men (88 %) and women (68 %). Intergenerational mobility out of the workforce was less common in men (8 %) than in women (27 %), while intragenerational mobility out of the workforce showed similar prevalences in men and women, irrespective of the categorization of workforce participation in childhood.

**Figure 3:**
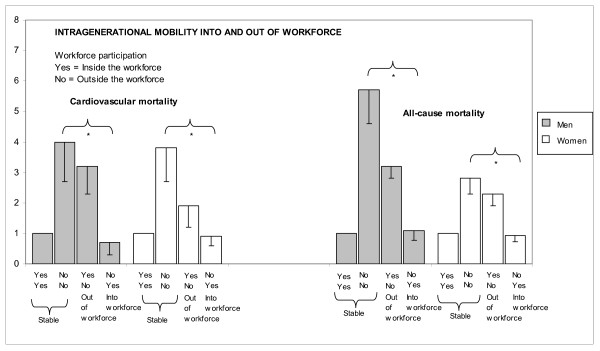
Age-adjusted HRRs for cardiovascular and all-cause mortality, by intragenerational mobility into and out of the workforce in Swedish men and women. The different categories at each bar denotes workforce participation at different periods in life – age 30–35 and at age 40–45. Those stable inside the workforce at age 30–35 and at age 40–45 were used as the reference. The lower border of the 95% confidence interval (CI) is marked.

Figure 3 shows the association between intragenerational social mobility into and out of the workforce and cardiovascular and all-cause mortality. The exact figures, with 95 % confidence intervals, can be seen in Table 8. Compared with subjects who were stable inside the workforce at the two stages in life, those who were stable outside the workforce showed a strongly increased cardiovascular and all-cause mortality, in both men (HRR = 4.0; 95% CI: 2.6, 6.0) and (HRR = 5.7; 95% CI: 4.5, 7.1), respectively, and women (HRR = 3.8; 95% CI: 2.6, 5.6) and (HRR = 2.8; 95% CI: 2.3, 3.5), respectively. Those who moved into the workforce lowered their mortality risk compared with subjects who were socially stable outside the workforce at the two periods in time (p < 0.05) and showed a HRR similar to that seen among men and women who were stable inside the workforce. Subjects who moved out of the workforce had strongly increased their mortality risk compared with subjects who were stable inside the workforce at both periods, in both men and women. As seen in Table 8, a similar pattern of association was seen with regard to intergenerational social mobility into and out of the workforce. The exception was subjects moving into the workforce who showed an increased HRR for cardiovascular and all-cause mortality in men and an increased cardiovascular mortality in women, compared with subjects who were inside the workforce at all three periods in life.

### Cumulative risk

#### Subjects having either manual or non-manual SEP at the three stages in life

**Figure 4:**
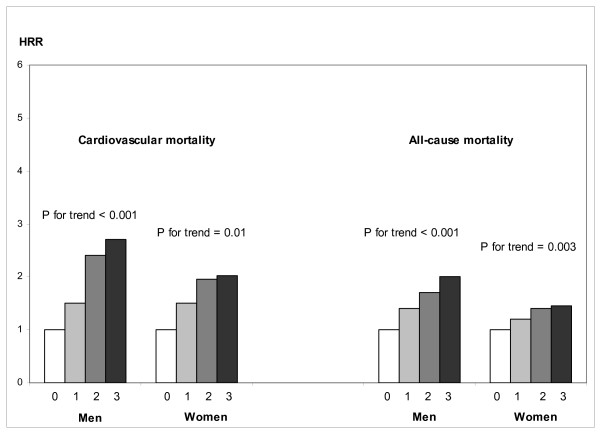
Bar graph showing age-adjusted HRRs of cardiovascular and all-cause mortality by a life course socioeconomic position (LCSEP) score ranging from 0 to 3 (combining childhood socioeconomic position, i.e., the SEP of the head of the household at ages 10–15, with the subject's own SEP at age 30–35 and at age 40–45: non-manual employees were given 0 points and manual workers were given 1 point). Only subjects having either a manual or non-manual SEP at the three stages in life were included in the analyses. P-values show p for trend in HRRs for cardiovascular and all-cause mortality. Subjects whose childhood SEP was categorized as non-manual and who had a non-manual occupation at age 30–35 and at age 40–45 were used as the reference group.

Figure 4 shows the cumulative effect of SEP on cardiovascular and all-cause mortality by use of a life course SEP (LCSEP) score in men and women. The exact figures, with 95 % confidence intervals, can be seen in Table 9. As shown in Figure 4, there was a clear upward trend in the mortality hazard rate ratios (HRR) with accumulated exposure to manual SEP in both men (p for trend < 0.001 for both cardiovascular and all-cause mortality) and women (p for trend = 0.01 for cardiovascular mortality) and (p for trend = 0.003 for all-cause mortality).

#### Whole population

**Figure 5:**
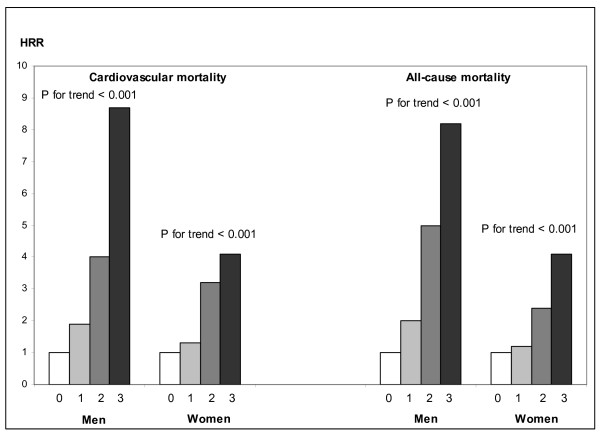
Bar graph showing age-adjusted HRRs for cardiovascular and all-cause mortality by the life course workforce participation (LCWFP) score ranging from 0 to 3, combining workforce participation of the head of the household during childhood at age 10–15 with the subject's own workforce participation at age 30–35 and at age 40–45. Subjects who were categorized as being inside the workforce were given 0 points, while subjects categorized as being outside the workforce were given 1 point. P-values show p for trend in HRRs for cardiovascular and all-cause mortality. Subjects whose head of the household during childhood was categorized as being inside the workforce and who themselves were inside the workforce at age 30–35 and at age 40–45 were used as the reference group.

A cumulative measure of workforce participation was taken by means of a life course workforce participation (LCWFP) score. As shown in Figure 5, there was a clear trend, in both men and women, for the HRR for cardiovascular and all-cause mortality to rise with an increased score (p for trend < 0.001). The exact figures, with 95 % confidence intervals, can be seen in Table 10.

### Statistical comparison of the three life course models

Using the Akaike Information Criterion (AIC) allowed us to compare the fit of the different models (the lower the AIC, the better the fit of the model). Among the working population there were very small differences (less than 5) in the AIC values between the different models, among both men and women. For example, among men, the AIC value was 5,815 for the critical period model, 5,811 for the social mobility model, and 5,814 for the cumulative risk model. In the whole population, the accumulation model showed a somewhat worse fit to the data (17 to 20 units higher AIC values) compared to the fit of the other two models.

## Discussion

The purpose of the present study was to gain a more complete picture of the relation between SEP and cardiovascular and all-cause mortality by taking a broad life course approach using different conceptual models. The relative importance of SEP at different periods in life, social mobility, and the cumulative risk, were analyzed in the same setting. In agreement with an earlier Swedish study on the association between SEP and cardiovascular morbidity [14], there was a strong relation between SEP and cardiovascular and all-cause mortality among those inside the workforce, irrespective of the conceptual model used. Furthermore, the results showed that being outside the workforce was associated with a strongly increased risk of future cardiovascular and all-cause mortality, in both men and women. When applying similar hypothetical models as used for those inside the workforce, it was shown that in both men and women, cardiovascular and all-cause mortality was affected by the totality of life course exposure to being outside the workforce. Moving out of the workforce increased the mortality risk, while moving into the workforce decreased the risk. While the former was true both between generations and within a working life, the latter was only true within a working life.

### The three life course socioeconomic models

#### Critical period

Several earlier studies have shown an association between early life SEP and mortality [1,5-7,9,12,18], while other studies could show no such associations [2,3]. Even though the importance of early life SEP has been the focus of the study of latent effects or critical periods of SEP on adult health, there may in theory also be stronger independent effects of SEP during certain periods, later in life, compared with others. In the study by Hallqvist et al. [14], the effect of having a manual SEP on CVD was found to be prominent at age 25–29. In our study, having a manual SEP was associated with an increased risk of future mortality compared with having a non-manual SEP at all the three studied periods in life. After mutual adjustment, the HRR for mortality was reduced for each period in life. Among men, there was still an independent effect at age 30–35 and age 40–45 with regard to cardiovascular as well as all-cause mortality, while an independent effect was seen among women at age 10–15 with regard to cardiovascular mortality. Subjects outside the workforce generally showed the highest mortality risk at all three age periods, with the strongest independent effect at age 40–45 in both men and women. Consequently, in our study, there was an independent effect of low SEP on future mortality, which was stronger at certain periods in life than at others.

#### Social mobility

Social mobility has been associated with health-related behaviors and psychosocial factors, as well as chronic diseases [27]. The causal direction of these associations is not completely evident. Some studies have shown an increased mortality risk among the upwardly mobile [19], but others have shown a decreased mortality risk [2,11]. Therefore, social mobility may be health-related, with disease causing downward social mobility. However, in our study, intragenerational mobility between a non-manual and a manual occupation was studied at the ages of 30–35 and 40–45. At this stage in life, the prevalence of CVD is very low. Furthermore, it is not evident that bad health goes with downward social mobility, since manual occupations, more often than non-manual occupations, require physically fit individuals [1]. Also, it has been argued that individual health problems have only a marginal effect on social mobility between different occupational status groups, but do affect mobility into and out of the workforce, both between generations and within working life [1,20]. In our study, while intragenerational as well as intergenerational downward social mobility was associated with an increased risk of future cardiovascular and overall death, the excess risk was much more pronounced among those who moved out of the workforce. Subjects moving out of the workforce were more often manual workers (46 %) than non-manual workers (40 %). Taken together, this suggests that even though social mobility may be health-related, it seems inadequate as an explanation for the association we found. The increased risk of future disease could also be a result of the mobility itself linked to stress-related factors, for example, or to lifestyle factors and the social network.

#### Cumulative risk

Several studies have shown a cumulative effect of SEP during the life course on adult mortality risk [4,5,8,10-12]. In a recent review by Pollitt et al., it was concluded that the cumulative life course model is more consistently supported than other models [16]. However, the authors also argued that it was difficult to compare the relative support for each model due to the different methodologic issues of each study design. In our study, there was a clear trend, in both men and women, for the HRR for cardiovascular and all-cause mortality to rise with an increasing number of periods of having a manual SEP. A similar, and even stronger, pattern of association was seen with regard to the totality of life course exposure to being outside the workforce.

### Comparison of the three life course socioeconomic models

Even though there is a strong correlation between the effect of each of these life course socioeconomic hypotheses on mortality, making it hard to separate the effects (e.g., the effect of social mobility out of the workforce per se from the effect of being outside the workforce), it is not obviously necessary to do so. The AIC showed a similar fit of the three different models among both men and women. In other words, one model cannot be said to be superior to the other models. The different models, or hypotheses, which the models are based on, can provide useful information, which can be combined to build a more complete picture of the relation between SEP and mortality, i.e., to see which exposures at different stages of life contribute to the development of adult disease. For example, exposure to manual occupations/being outside the workforce seems to be more important, with regard to cardiovascular and overall health, during certain periods in life and less important during other periods in life. These exposures also seem to add to the effect of each other, resulting in adult CVD as well as other types of disease. Furthermore, multiple exposures have an additive effect on cardiovascular and all-cause mortality from being exposed to manual occupations/being outside the workforce, irrespective of social mobility.

### Causal pathways

Regarding causal pathways between SEP and disease, the prevailing etiological model for adult chronic disease emphasizes adult risk factors. However, the importance of earlier life circumstances has attracted considerable attention during recent years. Atherosclerotic disease, respiratory-related diseases, and certain types of cancers are examples of diseases that develop throughout the life course [28]. For example, childhood SEP may influence the risk of future CVD through exposure to various psychosocial stressors (i.e., daily stress, poor family functioning, and social isolation) and can also indirectly affect adult health through its effect on adult psychosocial stressors, health-related behaviors, and adult SEP [29-31]. In adulthood, SEP has been shown to be related to material conditions (e.g., housing, work environment, family finances), as well as the social environment (e.g., working conditions, family situation, and social network) [32], which in turn may affect health-related behaviors and psychosocial stressors acting through biological mechanisms to cause cardiovascular events [28,33]. People outside the workforce have been shown to generally have more unfavourable life style habits and social networks compared with manual workers and also, compared with vocationally active individuals taken together [34]. These habits in turn increase their risk of future cardiovascular events. Finally, having a father outside the workforce may influence the possibilities of a person being vocationally active in adult life.

### Public health implications

Our study suggests that all three theoretical frameworks (i.e., critical periods, social mobility, and accumulation) are suitable for planning interventions to prevent premature mortality. Thus, a life course perspective seems to increase our understanding of the social causes of adult health and preventing strategies should be implemented with the aim of affecting individuals already in childhood. However, even though each model implies somewhat different interventions for avoiding premature mortality, the results of this study cannot say which theories best describe the links between life course SEP and mortality risk. In any case, it seems reasonable to conclude that societal initiatives for reducing deprivation and increasing participation in the work force across the life course is an investment that seems be compensated by increased health and reduced premature mortality

### Methodological issues

Certain methodological issues need to be addressed. First, misclassification of the end-point is a potential cause of bias. However, vital status at the end of the follow-up period was updated for all individuals by data linkage with the Swedish Causes of Death Register [22]. There is no reason to believe that incomplete retrieval of cases biased the results. Secondly, misclassification of exposure is a potential cause of bias. The classification of occupational status, as a measure of SEP, was based on information concerning current occupation. The SEI groups were categorized according to the criteria of Statistics Sweden [25], used for national demographic statistics. While the censuses of 1980 and 1990 used similar occupational categories, the classification scheme of the census of 1960 is somewhat less detailed, with fewer occupational categories and with more missing information. Therefore, we chose to use broad occupational categories, such as non-manual and manual occupations, to enable the longitudinal design of our study. Potential misclassification of SEP would be expected to be non-differential and would consequently lead to an underestimation of an effect on future cardiovascular and all-cause mortality. Furthermore, the longitudinal design allowed us to use multiple predictors of SEP over the life course, which would be expected to be associated with less measurement error than using single measures. In our study, we used the occupational status of the head of the family (i.e., in married couples the man's occupation) during upbringing as a proxy for childhood SEP. Since we used information from the census in 1960 where the father himself gave information on occupational status, this measure is not associated with recall bias as the use of retrospective measures may be. This measure was shown to be related to crowding in the household during childhood (defined as more than two inhabitants per room without counting the kitchen and one more room), since more of those with manual SEP (24 %) than non-manual SEP (7 %) had lived in a crowded household during childhood (data not shown). The occupational status of the father is thought to serve as a marker for environmental circumstances in childhood [35], validated by its association with adult height [27,32], and childhood material circumstances [36]. An alternative socioeconomic categorization for women would be the husband's socioeconomic group. However, a Swedish study on socioeconomic differences in myocardial infarction risk in Sweden compared use of the husband's occupation and use of the woman's own occupation as the basis for the socioeconomic classification, and the results showed similar incidence trends for the two measures [37].

One limitation of the study was that no information was available on presence of CVD at baseline. However, CVD under the age of 50 years is unusual and since bad health is not clearly associated with downward social mobility, this limitation ought not to affect the observed associations in any major way. In both men and women, those outside the workforce showed the highest risk of future cardiovascular mortality. However, only a few of these subjects were outside the workforce due to a disease. For example, at age 30–35, a total of 24 % of the women were outside the workforce. Out of these, 59 % were homemakers, 23 % were students, and only 6 % had a disability pension. At this age, the most common medical diagnoses in those receiving disability pension are not cardiovascular, but rather psychiatric and musculoskeletal. Furthermore, since we investigate premature cardiovascular mortality with the oldest subjects being 45 years of age at baseline investigation, the results are not prone to survivor bias in any major way.

One of the strengths of our study is that we had access to three decades of information on individual and household SEP. Moreover, our data cover the total general population in the county of Scania, which minimized the risk for selection bias. The mortality register encompass 97 % of all deaths in Sweden and the census participation rate varies between 98 % to 99 %. Therefore, the problem of many previous general population-based studies using population samples, with the healthiest people attending the study potentially attenuating the associations studied, is not present here.

## Conclusion

There was a strong relation between SEP and cardiovascular and all-cause mortality among those inside the workforce, irrespective of the conceptual model used, i.e., the critical period, social mobility, and accumulation models, when analyzed in the same setting. This suggests that a life course perspective increases our understanding of the social causes of adult health. The critical period, social mobility, and cumulative models showed the same fit to the data. That is, one model could not be pointed out as "the best" model. Consequently, although each model implies somewhat different interventions for avoiding premature mortality, even in this large un-selected sample it was not possible to adjudicate which theories best describe the links between life course SEP and mortality risk. Those outside the workforce generally had the highest mortality risk at all three age periods. When using the similar conceptual models based on workforce participation, it was shown that mortality risk was affected by accumulated exposure to being outside the workforce in both men and women. Moving out of the workforce increased mortality risk, while moving into the workforce decreased the risk. Thus, it seems reasonable to conclude that societal initiatives for increasing participation in the workforce is an investment that seems be compensated by increased health and reduced premature mortality.

## Competing interests

The author(s) declare that they have no competing interests.

## Authors' contributions

MR, BC, and JM were responsible for the conception, design, analysis, and interpretation of the data; the drafting, writing, and revision of the content; and the approval of the final version. JL and ML, both made substantial contributions to the design and interpretation of the data, the revision of the content, and the approval of the final version.

## Pre-publication history

The pre-publication history for this paper can be accessed here:



## Figures and Tables

**Table 1:** Description of the use of three conceptual life course models among those inside the workforce having a manual or non-manual socioeconomic position (SEP) at three periods in life (ages 10–15, 30–35 and 40–45). A total population investigation from Scania, Sweden.

Critical period*			Intergenerational (A,B,C)‡		Intragenerational (B,C)§
		
(A) 10–15 years	(B) 30–35 years	(C) 40–45 years	Social mobility†	Cumulative risk¶	Social mobility†
1	1	1	Stable down	3	Stable down
1	1	0	Upwards (C)	2	Upwards (C)
1	0	0	Upwards (B,C)	1	Stable up
0	1	1	Downwards (B,C)	2	
0	0	1	Downwards (C)	1	Downwards (C)
0	0	0	Stable up	0	

* Critical period model focuses on the importance of an independent effect of exposure to manual SEP during a specific sensitive period in life, having lasting effects on adult health. Non-manual workers = 0 and manual workers = 1.

† The social mobility model focuses on the importance of change in SEP to adult health.

‡ Intergenerational social mobility was defined as having a different socioeconomic position (SEP) in childhood than in adulthood. Intergenerational social mobility was defined as upward, downward or socially stable comparing the childhood SEP with the subject's own occupation at age 30–35 and at age 40–45.

§ Intragenerational social mobility was defined as having a different SEP at age 30–35 and at age 40–45. Intragenerational social mobility was defined as upward, downward or socially stable comparing the subject's own occupation at age 30–35 with the occupation at age 40–45.

¶ The cumulative risk model focuses on accumulation of risk during the life course. The cumulative score ranged from 0 to 3 since it was calculated by summing SEP values (i.e., non-manual employees were given 0 points and manual workers were given 1 point) at ages 10–15, 30–35 and 40–45.

**Table 2:** Description of the use of three conceptual life course models based on workforce participation at three periods in life (ages 10–15, 30–35 and 40–45). A total population investigation from Scania, Sweden.

Critical period*			Intergenerational (A,B,C)‡		Intragenerational (B,C)§
		
(A) 10–15 years	(B) 30–35 years	(C) 40–45 years	Social mobility†	Cumulative risk¶	Social mobility†
1	1	1	Stable outside workforce	3	Stable outside workforce
1	1	0	Into workforce (C)	2	Into workforce (C)
1	0	0	Into workforce (B,C)	1	Stable inside workforce
0	1	1	Out of workforce (B,C)	2	
0	0	1	Out of workforce (C)	1	Out of workforce (C)
0	0	0	Stable inside workforce	0	

* Critical period model focuses on the importance of an independent effect of exposure to being outside the workforce during a specific sensitive period in life, having lasting effects on adult health. Subjects inside the workforce = 0 and subjects outside the workforce = 1.

† The social mobility model focuses on the importance of change in workforce participation to adult health.

‡ Intergenerational social mobility was defined as having a different workforce participation in childhood than in adulthood. Intergenerational social mobility was defined as mobility out of, into, or stable inside or outside the workforce comparing the childhood workforce participation (i.e., workforce participation of the household) with the subject's own workforce participation at age 30–35 and at age 40–45.

§ Intragenerational social mobility was defined as having a different workforce participation at age 30–35 and at age 40–45. Intragenerational social mobility was defined as mobility out of, into, or stable inside or outside the workforce comparing the subject's own workforce participation at age 30–35 and at age 40–45.

¶ The cumulative risk model focuses on accumulation of risk during the life course. The cumulative score ranged from 0 to 3 since it was calculated by summing workforce participation values (i.e., subjects inside the workforce were given 0 points and subjects outside the workforce were given 1 point) at ages 10–15, 30–35 and 40–45.

**Table 3:** Age-adjusted hazard rate ratios (HRRs) of cardiovascular and all-cause mortality, by childhood socioeconomic position (SEP), adult SEP at age 30–35, and adult SEP at age 40–45 years in Swedish men.

			Cardiovascular mortality*					All-cause mortality				
				
				Age-adjusted		Mutually adjusted			Age-adjusted		Mutually adjusted	
								
	n	%	Cases	HRR‡	95% CI‡	HRR‡	95% CI‡	Cases	HRR‡	95% CI‡	HRR‡	95% CI‡
Childhood SEP†												
Non-manual§	10,882	27	125	1.0		1.0		318	1.0		1.0	
Manual	18,750	46	302	1.4	1.1, 1.7	1.1	0.9, 1.4	702	1.3	1.1, 1.5	1.1	1.0, 1.3
Self-employed	3,927	10	41	0.9	0.6, 1.3	0.8	0.6, 1.1	118	1.0	0.8, 1.3	0.9	0.7, 1.1
Farmers	5,346	13	64	1.0	0.8, 1.4	1.0	0.7, 1.3	157	1.0	0.8, 1.2	0.9	0.8, 1.2
Unclassified	361	1	6	1.5	0.7, 3.4	1.6	0.7, 3.7	12	1.2	0.7, 2.1	1.3	0.7, 2.3
Outside work force	1,706	4	40	2.0	1.4, 2.8	1.6	1.1, 2.3	84	1.7	1.3, 2.1	1.4	1.1, 1.7
Adult SEP age 30–35†												
Non-manual§	17,415	43	162	1.0		1.0		411	1.0		1.0	
Manual	17,612	43	325	2.0	1.7, 2.5	1.5	1.2, 1.9	706	1.7	1.5, 2.0	1.4	1.2, 1.7
Self-employed	3,199	8	49	1.6	1.2, 2.2	1.3	0.9, 1.8	127	1.7	1.4, 2.0	1.3	1.1, 1.6
Farmers	1,003	2	9	1.0	0.5, 1.9	1.4	0.6, 3.3	27	1.1	0.8, 1.7	1.3	0.8, 2.3
Unclassified	173	1	0	¶	¶	¶	¶	7	1.7	0.8, 3.7	1.3	0.6, 2.7
Outside work force	1,570	4	33	2.4	1.7, 3.5	1.3	0.9, 1.9	113	3.2	2.6, 4.0	1.8	1.4, 2.3
Adult SEP age 40–45†												
Non-manual§	18,673	46	161	1.0		1.0		416	1.0		1.0	
Manual	14,260	35	248	2.0	1.7, 2.5	1.5	1.2, 1.9	524	1.7	1.5, 1.9	1.3	1.1, 1.5
Self-employed	2,979	7	37	1.4	1.01, 2.1	1.2	0.9, 1.7	101	1.5	1.2, 1.9	1.3	1.03, 1.7
Farmers	968	2	5	0.6	0.2, 1.5	0.5	0.2, 1.5	20	0.9	0.6, 1.5	0.8	0.5, 1.5
Unclassified	1,605	4	27	2.0	1.3, 3.0	1.7	1.1, 2.6	73	2.0	1.6, 2.7	1.8	1.4, 2.3
Outside work force	2,487	6	100	4.9	3.8, 6.3	4.2	3.2, 5.5	257	4.9	4.2, 5.7	3.9	3.2, 4.6

* Cardiovascular mortality includes the following diagnoses; ICD 8: 390–459, ICD 9: 390–459 and ICD 10: I00-I99.

† Five categories among those inside the workforce: Non-manual, manual, self-employed, farmers and unclassified (inside the workforce although the work tasks are u and the category: Outside the work force (students, housewives, the unemployed, and disability pensioners). Childhood SEP was defined by SEP of the head of the ho primary occupation (in married couples i.e., the man's occupation).

‡ HRR, hazard rate ratio; CI, Confidence interval.

§ Reference category.

¶ Not applicable due to the low number of cases.

**Table 4:** Age-adjusted hazard rate ratios (HRRs) of cardiovascular and all-cause mortality, by childhood socioeconomic position (SEP), adult SEP at age 30–35, and adult SEP at age 40–45 years in Swedish women.

			Cardiovascular mortality*					All-cause mortality				
				
				Age-adjusted		Mutually adjusted			Age-adjusted		Mutually adjusted	
								
	n	%	Cases	HRR‡	95% CI‡	HRR‡	95% CI‡	Cases	HRR‡	95% CI‡	HRR‡	95% CI‡
Childhood SEP												
Non-manual§	10,578	26	46	1.0		1.0		244	1.0		1.0	
Manual	18,220	46	129	1.6	1.2, 2.3	1.5	1.04, 2.1	497	1.2	1.02, 1.4	1.1	0.9, 1.3
Self-employed	3,920	10	18	1.0	0.6, 1.8	1.0	0.6, 1.7	94	1.0	0.8, 1.3	1.0	0.8, 1.3
Farmers	4,979	13	23	1.0	0.6, 1.7	1.0	0.6, 1.6	97	0.8	0.7, 1.1	0.8	0.6, 1.3
Unclassified	316	1	3	2.2	0.7, 7.1	2.3	0.7, 7.3	9	1.3	0.6, 2.4	1.3	0.7, 2.5
Outside work force	1,664	4	17	2.3	1.3, 3.9	2.0	1.1, 3.4	53	1.3	1.0, 1.8	1.2	0.9, 1.6
Adult SEP ages 30–35 years												
Non-manual§	17,249	43	78	1.0		1.0		365	1.0		1.0	
Manual	10,857	27	79	1.6	1.2, 2.2	1.2	0.8, 1.8	315	1.4	1.2, 1.6	1.2	1.0, 1.5
Self-employed	1,159	3	5	0.9	0.4, 2.3	1.0	0.4, 2.5	27	1.1	0.7, 1.6	1.1	0.7, 1.6
Farmers	516	1	2	0.8	0.2, 3.4	0.6	0.1, 3.0	10	0.9	0.5, 1.7	0.8	0.4, 1.7
Unclassified	94	1	0	¶	¶	¶	¶	2	1.0	0.2, 4.0	0.9	0.2, 3.4
Outside work force	9,802	24	72	1.6	1.2, 2.2	1.1	0.8, 1.7	275	1.3	1.1, 1.6	1.1	0.9, 1.3
Adult SEP ages 40–45 years												
Non-manual§	20,442	52	87	1.0		1.0		416	1.0		1.0	
Manual	12,603	32	85	1.6	1.2, 2.1	1.3	0.9, 2.0	321	1.2	1.1, 1.4	1.1	0.9, 1.3
Self-employed	1,627	4	2	0.3	0.1, 1.2	0.3	0.1, 1.1	24	0.7	0.5, 1.1	0.7	0.4, 1.1
Farmers	372	1	2	1.3	0.3, 5.1	1.9	0.4, 9.5	7	0.9	0.4, 1.9	1.1	0.5, 2.7
Unclassified	1,292	3	11	2.1	1.1, 3.9	1.9	1.0, 3.6	38	1.5	1.1, 2.1	1.4	1.0, 1.9
Outside work force	3,341	8	49	3.5	2.5, 4.9	3.1	2.1, 4.6	188	2.8	2.4, 3.3	2.6	2.2, 3.2

* Cardiovascular mortality includes the following diagnoses; ICD 8: 390–459, ICD 9: 390–459 and ICD 10: I00-I99.

† Five categories among those inside the workforce: Non-manual, manual, self-employed, farmers and unclassified (inside the workforce although the work tasks are unknown) and the category: Outside the workforce (students, housewives, the unemployed, and disability pensioners). Childhood SEP was defined by SEP of the head of the household's primary occupation (in married couples i.e., the man's occupation).

‡ HRR, hazard rate ratio; CI, Confidence interval.

§ Reference category.

¶ Not applicable due to the low number of cases.

**Table 5:** Prevalences (%) of different trajectories among men and women having either a manual or non-manual socioeconomic position (SEP) at ages 10–15, 30–35 and 40–45. A total population investigation from Scania, Sweden.

Childhood SEP age 10–15†	Adult SEP age 30–35	Adult SEP age 40–45	Social mobility category*	Men n (%)	Women n (%)
Non-manual	Non-manual	Non-manual	IR S, IA S	5,778 (25.3)	5,447 (29.7)
Non-manual	Non-manual	Manual	IR D, IA D	239 (1.0)	190 (1.0)
Non-manual	Manual	Non-manual	IR D, IA U	635 (2.8)	369 (2.0)
Non-manual	Manual	Manual	IR D, IA S	1,635 (7.2)	1,067 (5.8)
Manual	Manual	Manual	IR S, IA S	7,348 (32.2)	4,573 (24.9)
Manual	Manual	Non-manual	IR U, IA U	1,601 (7.0)	832 (4.5)
Manual	Non-manual	Manual	IR U, IA D	456 (2.0)	302 (1.6)
Manual	Non-manual	Non-manual	IR U, IA S	5,112 (22.4)	5,580 (30.4)
*Total*				22,804 (100)	18,360 (100)

*IR D: Intergenerational downward mobility; IR U: Intergenerational upward mobility; IR S: Intergenerational stable; IA D: Intragenerational downward mobility; IA U: Intragenerational upward mobility; IA S: Intragenerational stable.

†Childhood SEP was defined by SEP of the head of the household's primary occupation (in married couples i.e., the man's occupation)

**Table 6:** Age-adjusted hazard rate ratios (HRRs) of cardiovascular and all-cause mortality, by inter- and intragenerational social mobility in men and women having either a manual or non-manual socioeconomic position (SEP) at ages 10–15, 30–35, and 40–45. A total population investigation from Scania, Sweden.

	Cardiovascular mortality*				All-cause mortality			
		
	Men		Women		Men		Women	
				
	Age-adjusted		Age-adjusted		Age-adjusted		Age-adjusted	
				
	HRR†	95% CI†	HRR†	95% CI†	HRR†	95% CI†	HRR†	95% CI†
Intergenerational social mobility‡								
Stable non-manual¶	1.0		1.0		1.0		1.0	
Stable manual	2.7	1.9, 3.8	2.0	1.1, 3.5	2.0	1.6, 2.5	1.4	1.1, 1.9
Downward mobile	2.4	1.5, 3.7	1.4	0.6, 3.3	1.7	1.3, 2.3	1.5	1.1, 2.2
Upward mobile	1.6	1.1, 2.4	1.6	1.0, 2.9	1.4	1.1, 1.8	1.2	0.9, 1.5
Intragenerational social mobility§								
Stable non-manual¶	1.0		1.0		1.0		1.0	
Stable manual	2.3	1.7, 3.0	1.5	1.0, 2.3	1.7	1.4, 2.0	1.4	1.1, 1.7
Downward mobile	2.9	1.7, 4.9	1.9	0.7, 5.2	1.8	1.3, 2.7	1.6	1.0, 2.7
Upward mobile	1.8	1.2, 2.8	1.7	0.9, 3.5	1.4	1.1, 1.9	1.3	0.9, 1.9

* Cardiovascular mortality includes the following diagnoses; ICD 8: 390–459, ICD 9: 390–459 and ICD 10: I00-I99.

† HRR, hazard rate ratio; CI, Confidence interval.

‡ Intergenerational social mobility was defined as having a different socioeconomic position (SEP) in childhood than in adulthood. Intergenerational social mobility was defined as upward, downward or socially stable comparing the childhood SEP with the subject's own occupation at age 30–35 and at age 40–45. Those stable non-manual in childhood, at age 30–35 and at age 40–45 were used as the reference.

§ Intragenerational social mobility was defined as having a different SEP at age 30–35 and at age 40–45. Intragenerational social mobility was defined as upward, downward or socially stable comparing the subject's own occupation at age 30–35 with the occupation at age 40–45. Those stable non-manual at age 30–35 and at age 40–45 were used as the reference.

¶ Reference category.

**Table 7:** Prevalences (%) of different trajectories in men and women, by workforce participation at ages 10–15, 30–35 and 40–45. A total population investigation from Scania, Sweden.

Childhood workforce participation age 10–15†	Adult workforce participation age 30–35	Adult workforce participation age 40–45	Social mobility category*	Men n (%)	Women n (%)
Inside	Inside	Inside	IR S, IA S	35,951 (87.7)	27,086 (68.3)
Inside	Inside	Outside	IR O, IA O	1,850 (4.5)	1,583 (4.0)
Inside	Outside	Inside	IR O, IA I	980 (2.4)	7,783 (19.6)
Inside	Outside	Outside	IR O, IA S	485 (1.2)	1,561 (3.9)
Outside	Outside	Outside	IR S, IA S	42 (0.1)	101 (0.3)
Outside	Outside	Inside	IR I, IA I	63 (0.2)	357 (0.9)
Outside	Inside	Outside	IR I, IA O	110 (0.3)	96 (0.2)
Outside	Inside	Inside	IR I, IA S	1,491 (3.6)	1,110 (2.8)
*Total*				40,972 (100)	39,677 (100)

*IR O: Intergenerational mobility out of the workforce; IR I: Intergenerational mobility into the workforce; IR S: Intergenerational stable; IA O: Intragenerational mobility out of the workforce; IA I: Intragenerational mobility into the workforce; IA S: Intragenerational stable.

†Childhood workforce participation was defined by workforce participation of the household.

**Table 8:** Age-adjusted hazard rate ratios (HRRs) of cardiovascular and all-cause mortality, by inter- and intragenerational social mobility into and out of the workforce at ages 10–15, 30–35, and 40–45. A total population investigation from Scania, Sweden.

	Cardiovascular mortality*				All-cause mortality			
		
	Men		Women		Men		Women	
				
	Age-adjusted		Age-adjusted		Age-adjusted		Age-adjusted	
				
	HRR†	95% CI†	HRR†	95% CI†	HRR†	95% CI†	HRR†	95% CI†
Intergenerational social mobility‡								
Stable inside the workforce¶	1.0		1.0		1.0		1.0	
Stable outside the workforce	8.7	3.2, 23.2	4.1	1.01, 16.5	8.2	4.3, 15.9	4.1	2.1, 7.9
Out of the workforce	2.4	2.0, 3.0	1.6	1.2, 2.1	2.8	2.5, 3.3	1.4	1.2, 1.6
Into the workforce	1.7	1.2, 2.4	1.8	1.1, 3.2	1.5	1.2, 1.9	1.2	0.9, 1.7
Intragenerational social mobility§								
Stable inside the workforce¶	1.0		1.0		1.0		1.0	
Stable outside the workforce	4.0	2.6, 6.0	3.8	2.6, 5.6	5.7	4.5, 7.1	2.8	2.3, 3.5
Out of the workforce	3.2	2.5, 4.1	1.9	1.1, 3.1	3.2	2.7, 3.7	2.3	1.8, 2.8
Into the workforce	0.7	0.4, 1.4	0.9	0.6, 1.3	1.1	0.8, 1.5	0.9	0.8, 1.1

* Cardiovascular mortality includes the following diagnoses; ICD 8: 390–459, ICD 9: 390–459 and ICD 10: I00-I99.

† HRR, hazard rate ratio; CI, Confidence interval.

‡ Intergenerational social mobility into and out of the workforce was defined as having a different workforce participation in childhood than in adulthood. Those stable inside the workforce in childhood (defined by workforce participation of the household), at age 30–35 and at age 40–45 were used as the reference.

§ Intragenerational social mobility was defined as having a different workforce participation between age 30–35 and at age 40–45. Those stable inside the workforce at age 30–35 and at age 40–45 were used as the reference.

¶ Reference category.

**Table 9:** Age-adjusted hazard rate ratios (HRRs) of cardiovascular and all-cause mortality, by the life course socioeconomic position (LCSEP) score in men and women having either a manual or non-manual socioeconomic position (SEP) at ages 10–15, 30–35, and 40–45. A total population investigation from Scania, Sweden.

	Cardiovascular mortality*				All-cause mortality			
		
	Men		Women		Men		Women	
				
	Age-adjusted		Age-adjusted		Age-adjusted		Age-adjusted	
				
	HRR†	95% CI†	HRR†	95% CI†	HRR†	95% CI†	HRR†	95% CI†
Life course socioeconomic position (LCSEP) score‡								
0§	1.0		1.0		1.0		1.0	
1	1.5	1.0, 2.2	1.5	0.9, 2.7	1.4	1.1, 1.7	1.2	0.9, 1.5
2	2.4	1.6, 3.6	2.0	1.02, 3.9	1.7	1.3, 2.2	1.4	1.05, 2.0
3	2.7	1.9, 3.8	2.0	1.1, 3.5	2.0	1.6, 2.5	1.5	1.1, 1.9
								
*p for trend*	<0.001		0.01		<0.001		0.003	

* Cardiovascular mortality includes the following diagnoses; ICD 8: 390–459, ICD 9: 390–459 and ICD 10: I00-I99.

† HRR, hazard rate ratio; CI, Confidence interval.

‡ The life course socioeconomic position (LCSEP) score ranges from 0 to 3, and is a combination of childhood socioeconomic position at age 10–15 (based on the head of the household's primary occupation) and the subject's socioeconomic position at age 30–35 and 40–45. Non-manual employees were given 0 points and manual workers were given 1 point. Subjects having a non-manual socioeconomic position at all three periods in time were used as the reference group.

§ Reference category.

**Table 10:** Age-adjusted hazard rate ratios (HRRs) of cardiovascular and all-cause mortality, by the life course workforce participation (LCWFP) score in men and women at ages 10–15, 30–35, and 40–45. A total population investigation from Scania, Sweden.

	Cardiovascular mortality*				All-cause mortality			
		
	Men		Women		Men		Women	
				
	Age-adjusted		Age-adjusted		Age-adjusted		Age-adjusted	
				
	HRR†	95% CI†	HRR†	95% CI†	HRR†	95% CI†	HRR†	95% CI†
Life course workforce participation (LCWFP) score‡								
0§	1.0		1.0		1.0		1.0	
1	1.9	1.6, 2.4	1.3	1.0, 1.8	2.0	1.7, 2.3	1.2	1.03, 1.4
2	4.0	2.7, 5.7	3.2	2.2, 4.8	5.0	4.0, 6.2	2.4	1.9, 2.9
3	8.7	3.2, 23.2	4.1	1.01, 16.4	8.2	4.3, 15.9	4.1	2.1, 7.9
								
*p for trend*	< 0.001		< 0.001		< 0.001		< 0.001	

* Cardiovascular mortality includes the following diagnoses; ICD 8: 390–459, ICD 9: 390–459 and ICD 10: I00-I99.

† HRR, odds ratio; CI, Confidence interval.

‡ The life course workforce participation (LCWFP) score ranges from 0 to 3, and is a combination of childhood workforce participation at age 10–15 (i.e., the head of the household's workforce participation) and the subject's workforce participation at age 30–35 and 40–45. Those inside the workforce were given 0 points and those outside the workforce were given 1 point. Those inside the workforce at all three periods in time were used as the reference group.

§ Reference category.
